# Primary cardiac angiosarcoma: a case report

**DOI:** 10.3389/fonc.2025.1575207

**Published:** 2025-05-29

**Authors:** Yin Yuan, Zhao Yu, Wenjie Diao, Zelai He, Jianming Ding, Gengming Wang

**Affiliations:** ^1^ Department of Radiation Oncology, The First Affiliated Hospital of Bengbu Medical University, Bengbu, China; ^2^ Department of Cardiovascular Surgery, The First Affiliated Hospital of Bengbu Medical University, Bengbu, China

**Keywords:** primary cardiac angiosarcoma, surgery, cardiac tumor, chemotherapy, multidisciplinary team

## Abstract

Primary cardiac tumors are extremely rare, with an incidence rate ranging from only 0.17% to 0.33%. Clinically, their diagnosis is often delayed due to nonspecific clinical symptoms. This paper reports a case of primary cardiac angiosarcoma (PCAS) pathologically confirmed via surgery, presenting with recurrent palpitations during exertion as the initial symptom. The patient subsequently developed bilateral lung and liver metastases and received chemotherapy. Through a comprehensive review of the literature, this report explores the diagnostic and therapeutic strategies for PCAS, with a focus on analyzing the mechanisms underlying the atypical disease course, the rationale for treatment decisions, and the key clinical insights.

## Case study introduction

The case of Patient L (with informed consent obtained) provides a compelling account that highlights the complexity and highly aggressive nature of cardiac angiosarcoma. Through in-depth analysis of this case and a systematic review of current diagnostic and treatment strategies, this study aims to enhance the medical community’s clinical awareness of PCAS and underscore the critical importance of recognizing this highly aggressive disease.

### Patient background

Patient L, a 40-year-old male diagnosed with PCAS, developed distant metastases shortly after resection of the primary tumor. Following regular chemotherapy, pulmonary lesions were well-controlled, but newly developed metastatic sarcoma lesions emerged in the liver.

### Clinical profile

Weight: 75kg; BMI: 24.49 kg/m^2^.

KPS score: 80 points;NRS score: 2 points.

Psychosocial status is Stable.

Denies any significant past medical history or family history of hereditary diseases.

### Treatment course

**Figure 1:**
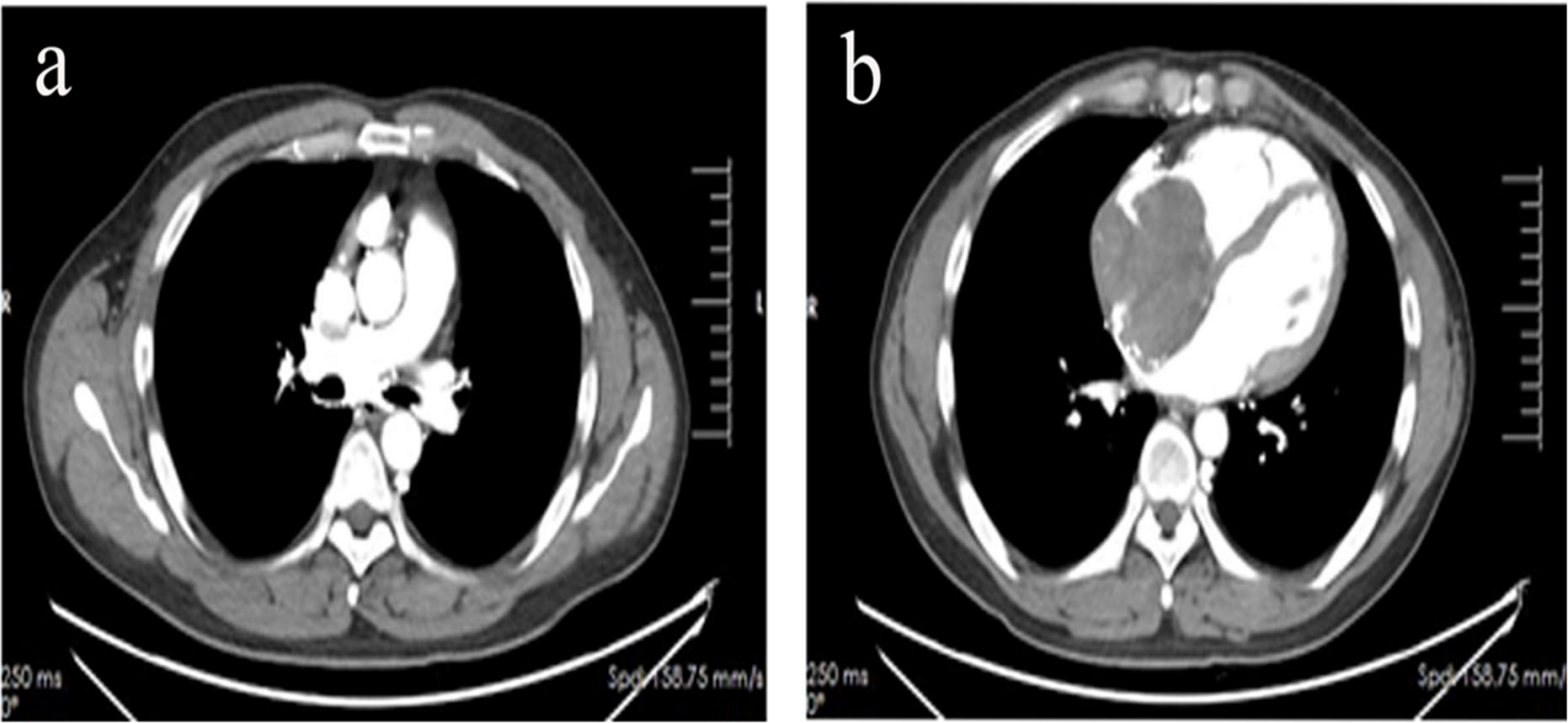
CT venous angiography revealed filling defects in the superior vena cava **(a)** and right atrium **(b)**.

In March 2024, the patient presented with a chief complaint of recurrent palpitations after exertion for over two months. Transthoracic echocardiography revealed a solid echo in the right atrium, with the initial diagnosis considering possible cardiac myxoma. Aortic CTA in April 2024 revealed mixed plaques in the lower aorta and left common iliac artery, along with a space-occupying lesion in the right atrium; CT venography (**Figure 1**) demonstrated filling defects in the right atrium and superior vena cava, highly suspicious of a neoplastic lesion. After completing preoperative evaluations and excluding contraindications, the patient underwent right atrial tumor resection, right atrium reconstruction, and superior vena cava thrombectomy via median sternotomy under general anesthesia with cardiopulmonary bypass. Postoperatively, a permanent pacemaker was implanted due to sick sinus syndrome. The surgical procedure was uneventful. Postoperative imaging and laboratory examinations showed no residual tumor or distant metastases.

**Figure 2:**
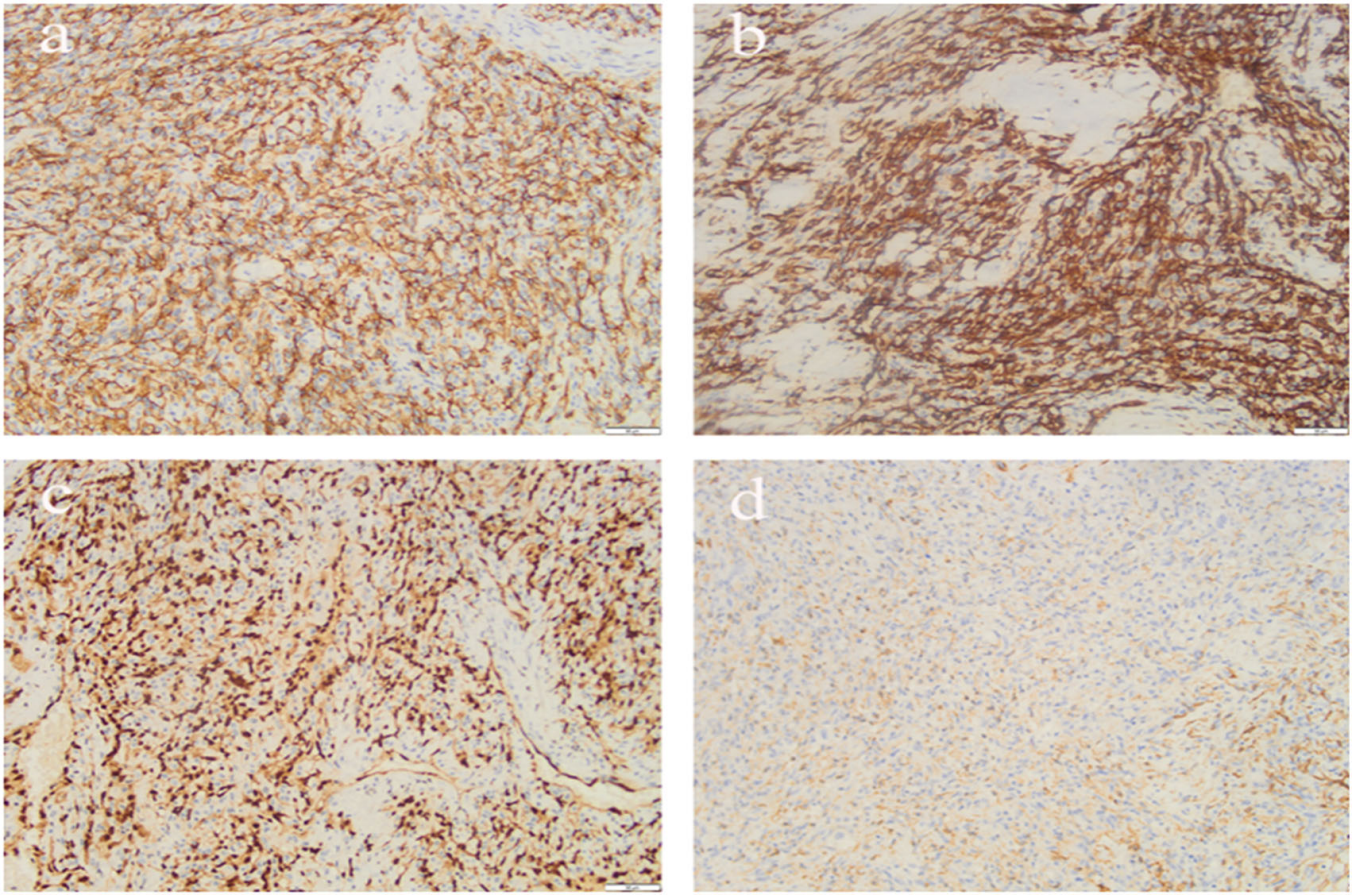
Immunohistochemistry of angiosarcoma of pericardium. [**(a)** CD34 **(b)** CD31 **(c)** ERG **(d)** SMA].

Postoperative pathology revealed a right atrial tumor with extensive hemorrhage and necrosis, measuring up to 11.0 cm in maximum diameter.Immunohistochemical staining (**Figure 2**) confirmed features consistent with angiosarcoma: tumor cells were positive for CD34, CD31, ERG, and FIL-1, negative for CK and SMA, with a KI-67 proliferation index of approximately 25%. The final diagnosis was primary angiosarcoma of the right atrium.

Imaging follow-up in June 2024 suggested possible metastases in both lungs and the liver. Given the disease progression and absence of chemotherapy contraindications, the patient first received one cycle of chemotherapy with the regimen “Ifosfamide 2.5g + Epirubicin hydrochloride 140mg.” Due to drug supply issues, the regimen was later adjusted to “Ifosfamide 2.5g + Doxorubicin hydrochloride liposome 35mg,” with a total of four cycles completed. During treatment, the patient developed cardiac toxicity, myelosuppression, and gastrointestinal reactions, all of which resolved after symptomatic management. The overall chemotherapy course was safe and well-tolerated.

**Figure 3:**
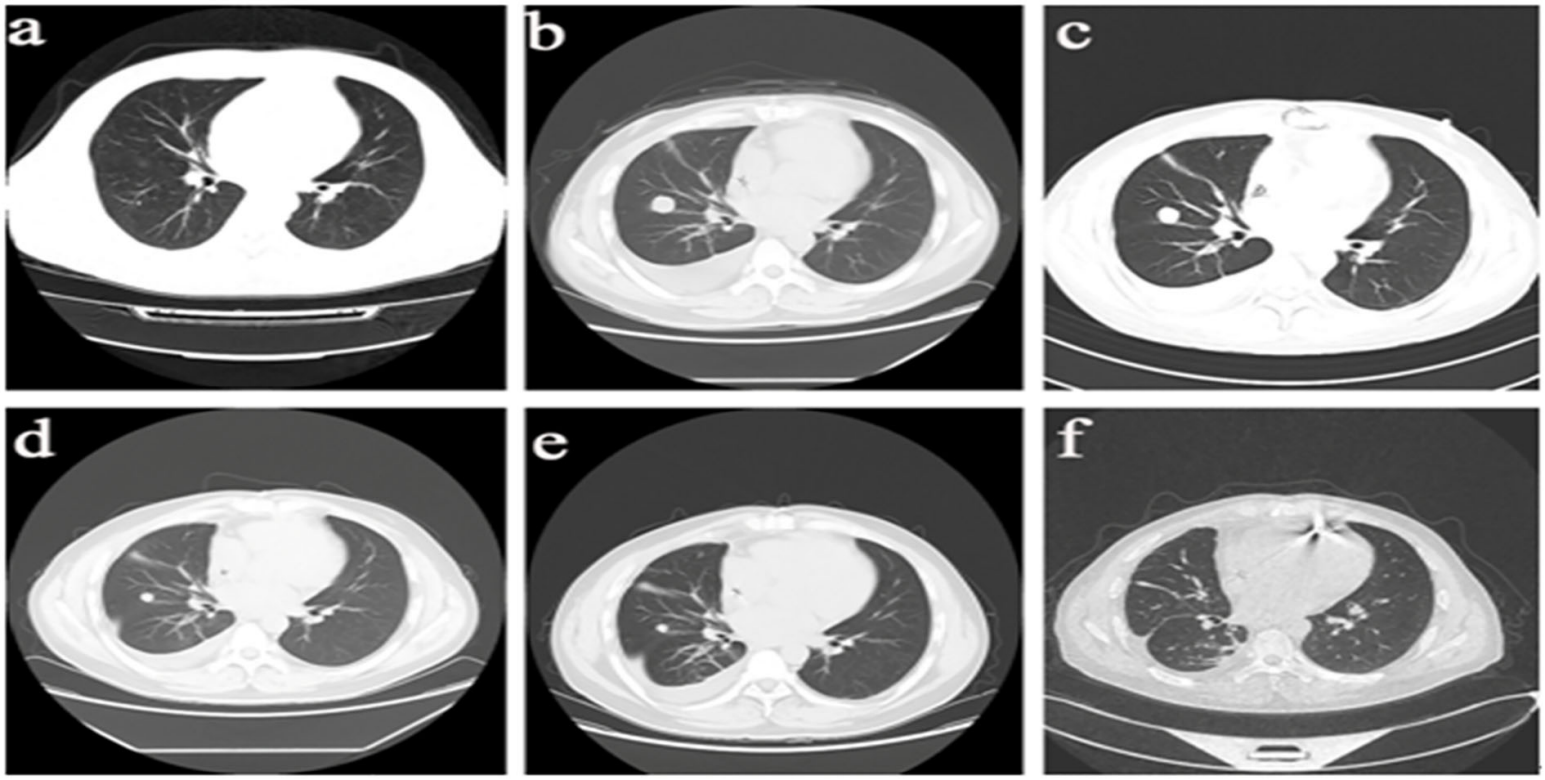
Imaging Progression of Pulmonary Lesions in the Patient. **(a)** March 29, 2024: Scattered ground-glass and nodular high-density opacities in both hangs, with the longest diameter measuring 4-7 mm. Bilateral hilar shadows are not enlarged. **(b)** June 12, 2024: Scattered nodules in both lungs, with some nodules showing interval enlargement compared to prior imaging. The largest nodule measures approximately 20mm×19mm, demonstrating lobulation and pleural indentation signs, with contrast enhancement observed onpost-contrast imaging. **(c)** July 25, 2024: A roundish soft tissue density opacity measuring approximately 16mm in diameter is noted adjacent to the right oblique fissure, demonstrating mild enhancement on contrast-enhanced scan, suspicious for metastasis. **(d)** October 24, 2024: Scattered patchy and linear opacities in the right lung, with a solid small nodule adjacent to the right oblique fissure measuring approximately 12×11mm. Enhancement is noted on imaging, and regular follow-up is advised. **(e)** December 4, 2024: A solid small nodule adjacent to the right oblique fissure demonstrates slight reduction in size compared to previous imaging, now measuring approximately 10×9mm. Enhancement is noted, and bilateral hilar shadows remain unenlarged. **(f)** March 15, 2025: Afew solid nodules are noted in the right middle lobe and left lower lobe of the lung, with the largest one measuring approximately 7x4mm.

Regular follow-up examinations revealed that chest CT (**Figure 3**) indicated good control of pulmonary lesions, while upper abdominal CT showed a large space-occupying lesion measuring approximately 14 cm in the right hepatic lobe, confirming the development of hepatic metastatic sarcoma. The patient is currently scheduled to undergo TACE, with subsequent close clinical follow-up.

### Follow-up and lifestyle changes

The patient’s physical performance status and quality of life have significantly declined. He reports frequent dyspnea and palpitations after exertion, accompanied by paroxysmal chest tightness lasting 5–10 minutes. His ability for daily activities is limited by the condition.

### Treatment journey

See **Figure 4**.

**Figure 4:**
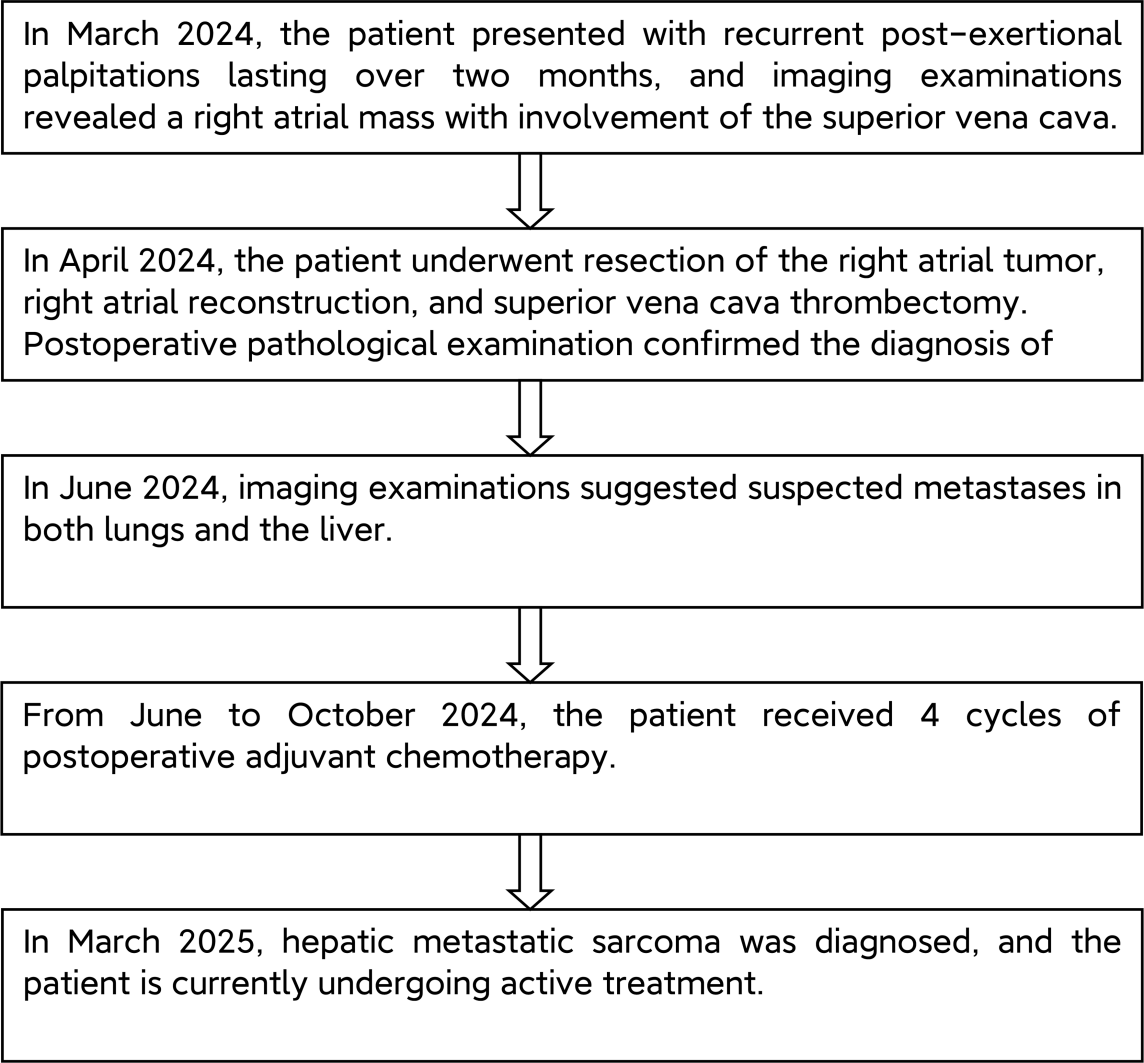
Overview of the Patient Treatment Process Flowchart.

## Discussion

Angiosarcoma is an extremely rare and highly aggressive soft tissue sarcoma, arising primarily from endothelial cells of blood or lymphatic vessels, with the potential to occur in various organs and soft tissues throughout the body (1). PCAS, the most common histological type among primary malignant cardiac tumors, accounts for approximately one-third of such neoplasms and is most commonly found in the right atrium (2),which not only shows aggressive growth to the surrounding myocardium, but also can invade the inferior vena cava and tricuspid valve (3). Review of the literature reveals that cardiac angiosarcoma can occur across all age groups, though it predominantly affects males under 65 years of age (4). PCAS is rare in clinic, has poor prognosis, lack of specificity in clinical manifestations, is not easy to distinguish from other diseases, and obvious clinical symptoms appear late. Most of the symptoms are dyspnea, chest pain, heart failure, palpitations, syncope, but also vena cava obstruction, pulmonary embolism, hemoptysis and so on (5, 6). Studies (7) found that about 66% to 89% of patients had lung, liver, bone and other distant metastases at the first diagnosis, and the lung was the most common site of metastasis. In this case, the patient presented with superior vena cava obstruction at initial diagnosis, demonstrated intraoperative invasion of the right atrial surface by the tumor, and developed subsequent metastases to both lungs and the liver—all clinical features consistent with previous literature reports.

At present, the detection of the disease mainly relies on imaging tests, including echocardiography, CT, MRI and PET/CT. Among them, echocardiography is the primary choice for clinical detection of cardiac angiosarcoma. Transthoracic echocardiography can determine tumor size, anatomic location and valve abnormalities, and its sensitivity to detect cardiac tumors can reach 97% (8, 9). However, echocardiography can not determine the nature of the tumor, and CT, MRI, and PET/CT are needed to help diagnose the tumor shape and the presence of metastasis. Relevant literature has shown that endocardial myocardial biopsy can also be used for the diagnosis of angiosarcoma (10), but endocardial biopsy has a high risk of bleeding and a high false negative rate. Therefore, for patients who are highly suspected of angiosarcoma and can be operated on clinically, direct surgical treatment and obtaining pathological results are recommended. This case highlights the need for a high index of suspicion for PCAS in middle-aged males presenting with exertional palpitations combined with vena cava obstruction, advocating for early integration of imaging and pathological confirmation to avoid misdiagnosis as cardiac myxoma (11).

For PCAS, established treatment protocols remain undefined. Most domestic and international studies favor multidisciplinary approaches integrating systemic therapies (surgery, chemotherapy, targeted therapy, immunotherapy) and radiotherapy (12). However, the role of chemotherapy or radiotherapy in its treatment has not been proven to be beneficial, and complete surgical resection is the only treatment that has been shown to extend survival (13). Given the patient’s good physical performance status and sufficient operative tolerance, surgical resection was selected as the primary antineoplastic therapy.According to relevant literature reports, the average survival time of patients without surgical resection is 3.8 ± 2.5 months, and the postoperative survival time of patients with surgical treatment is 2–55 months (14);Meanwhile, another retrospective study (15) also showed that patients with negative surgical margins had a longer median overall survival than those with positive surgical margins. However, due to the deep anatomical location, proximity to vital vascular structures, and highly aggressive biological behavior of PCAS, patients often face a high risk of local recurrence or distant metastasis after surgery. In this case, the patient developed hepatic and pulmonary metastases shortly after resection. Although combined postoperative radiotherapy and chemotherapy were initially planned, the use of radiotherapy was limited by the low radiation tolerance of cardiac tissue (16). Concomitant with the presence of distant metastases, systemic palliative chemotherapy was thus selected as the subsequent treatment strategy. In recent years, studies have shown that paclitaxel drugs have good anti-angiogenic activity, and can significantly improve the survival rate of patients in the treatment of angiosarcoma (17). At present, the relevant reports at home and abroad have used platinum, doxorubicin, paclitaxel, methotrexate and other cytotoxic drugs as common drugs for patients with cardiovascular angiosarcoma (18, 19).

At the same time, with the breakthrough of immunotherapy, especially programmed cell death receptor-1 (PD-1) and programmed cell death ligand (PD-L1) in tumor treatment, immunotherapy has become another research hotspot in the treatment of tumor. Relevant studies (20) have shown that the prognosis of patients with hemangiosarcoma is significantly correlated with the expression of PD-1 and PD-L1, suggesting that the potential of immunotherapy in the treatment of this disease deserves attention. On the other hand, more and more studies have been published on the significant efficacy of anti-tumor targeted drugs in the treatment of soft tissue sarcoma. For example, the ALTER0203 study (21) found that the progression-free survival of soft tissue sarcoma was significantly prolonged in the anlotinib group compared with the placebo group (6.27 vs 1.47 months, P < 0.001). It provides strong evidence-based medical evidence for anlotinib to become a grade I recommendation of second-line drug for CSCO soft tissue sarcoma. Zhu Bingjing et al. also reported a patient who was treated with the oral anti-angiogenesis targeting drug “anlotinib” and showed significant improvement in clinical symptoms (22). At the same time, Zhao Xin et al. performed chemotherapy combined with immunotherapy for a patient with cardiac angiosarcoma, and reexamination indicated that the lesion was stable after 2 cycles of treatment with the “paclitaxel (albumin-binding type)+ carboplatin + pembrolizumab” regimen, and no significant progress was observed in the patient’s condition (23). In addition, new treatments including heart transplantation and autologous transplantation after surgical resection have been reported, but with poor results.

According to the 2024 consensus on the diagnosis and treatment of rare cardiac tumors by the European Association for Cardio-Thoracic Surgery (EACTS), for oligometastatic PCAS, local therapies such as TACE and radiotherapy are recommended to control symptoms, with anthracycline-based systemic chemotherapy as the first-line regimen, while novel targeted therapy and immunotherapy are suggested to be incorporated into clinical trials (12). The decision not to attempt immunotherapy or targeted therapy in this case was based on the following clinical considerations: First, the patient developed myelosuppression during chemotherapy, and immune checkpoint inhibitors (such as PD-1 inhibitors) may significantly increase the risk of immune-related pneumonia in patients with lung metastases, necessitating careful evaluation of the benefit-risk ratio; Second, although studies have confirmed that anlotinib significantly prolongs progression-free survival in soft tissue sarcomas (21), its efficacy in cardiac angiosarcoma remains unclear, and its potential risk of QT interval prolongation may complicate the management of postoperative pacemaker dependency (22); Third, the hypervascular characteristics of the hepatic metastases made local therapies like TACE more suitable for precise intervention targeting tumor biology compared to systemic therapy. Integrating consensus recommendations, individual tolerance, and tumor pathophysiological characteristics, novel targeted or immunotherapies were not applied in this case.

The relatively stable clinical course after metastasis, differing from the typical rapidly fatal course of PCAS, can be explained by three potential mechanisms: First, the synergy between chemotherapy efficacy and tumor biology. The “anthracycline (liposomal doxorubicin) combined with ifosfamide” regimen used here, as a first-line chemotherapy for soft tissue sarcomas (including angiosarcoma) (18), induced drug-related cardiotoxicity indicating that drug concentrations reached therapeutic levels, indirectly reflecting potential inhibition of tumor cell proliferation (17); the tumor’s Ki-67 proliferation index of 25% (moderate malignancy) suggested moderate cell cycle activity and potential sensitivity to anthracyclines acting on the S phase (19). Second, organ-specific microenvironments of metastases influenced tumor behavior: hepatic metastases, facilitated by abundant blood supply promoting angiogenesis, showed rapid invasive growth (growing to 14 cm in diameter within 3 months), while pulmonary metastases remained stable due to the inhibitory effects of the lung microenvironment on tumor cell adhesion and proliferation (7). Additionally, although PD-1/PD-L1 testing was not performed, recent studies have shown that PD-L1 expression in angiosarcoma correlates with prognosis, and chemotherapy may activate host anti-tumor immune responses by inducing tumor cell necrosis, releasing tumor-associated antigens and danger signals (20), thereby remodeling the tumor immune microenvironment and providing a potential immunological mechanism for stage-specific disease control.

## Conclusion

This case, managed through a multidisciplinary team (MDT) approach throughout the care continuum, profoundly illustrates the aggressive biological characteristics and diagnostic-treatment challenges of PCAS. The core insights are as follows:

1. Early precise identification of high-risk populations: In males aged 40–65 years presenting with atypical symptoms such as exertional palpitations combined with superior vena cava obstruction (e.g., facial edema, jugular venous distension), PCAS should be highly suspected. Early confirmation requires integration of transthoracic echocardiography, contrast-enhanced CT/MRI, and pathological biopsy.
2. Surgery-centric comprehensive treatment strategy: Complete surgical resection remains the only proven intervention to prolong survival. Postoperative adjuvant chemotherapy based on anthracyclines can delay metastatic progression but necessitates close monitoring of anthracycline-related cardiotoxicity and myelosuppression.
3. Stratified intervention for recurrent/metastatic lesions: For oligometastatic lesions (≤3 organs), a “local control priority” strategy is recommended, combining local therapies (e.g., TACE, stereotactic radiotherapy) with systemic chemotherapy. For widely metastatic cases, immunotherapy or targeted therapy should be considered within standardized clinical trials following MDT assessment of cardiac function and bone marrow reserve.
4. Enhanced MDT collaboration and clinical vigilance: MDT collaboration involving cardiothoracic surgery, medical oncology, radiology, and interventional disciplines is pivotal for optimizing individualized treatment. Meanwhile, heightened clinical awareness of atypical symptoms is essential to overcome diagnostic delays caused by the insidious presentation of PCAS.

### Clinical key points

1. Early screening strategy for high-risk populations

In males aged 40–65 years with unexplained exertional palpitations, progressive dyspnea, or superior vena cava syndrome (e.g., facial swelling, chest wall varicose veins):

- Prioritize transthoracic echocardiography to initially evaluate the location, size, and adjacency of right heart masses.
- Combine with contrast-enhanced CT/MRI to further define tumor invasion extent and metastatic status, avoiding missed diagnosis of malignant tumors due to nonspecific symptoms.

2. Preferred pathway for pathological confirmation

Given the high bleeding risk and false-negative rate of endomyocardial biopsy, direct surgical resection for pathological diagnosis is recommended for suspected patients with good performance status (KPS ≥70) and no absolute surgical contraindications. This approach circumvents the limitations of invasive biopsy and provides precise histological evidence for subsequent treatment.

3. Stratified treatment strategy for metastatic lesions

- Oligometastasis (single or 2–3 organ metastases): Prioritize local therapies (e.g., TACE, radiotherapy) to control dominant lesions, combined with anthracycline-based systemic chemotherapy to address both local tumor control and systemic micrometastases.
- Widespread metastasis (≥4 organs or multiple metastatic foci): Following comprehensive MDT evaluation of cardiac function, bone marrow reserve, and comorbidities (e.g., pacemaker dependency), consider enrollment in clinical trials for immunotherapy (e.g., PD-1 inhibitors) or targeted therapy (e.g., anlotinib). Treatment protocols must undergo MDT review to ensure a balance between safety and potential benefits.

## Data Availability

The original contributions presented in the study are included in the article/Supplementary Material. Further inquiries can be directed to the corresponding author/s.
